# Macrophage‐Derived Extracellular Vesicles‐Coated Palladium Nanoformulations Modulate Inflammatory and Immune Homeostasis for Targeting Therapy of Ulcerative Colitis

**DOI:** 10.1002/advs.202304002

**Published:** 2023-10-09

**Authors:** Jiahui Cheng, Yiming Zhang, Liang Ma, Wenxian Du, Qiang Zhang, Rifeng Gao, Xinxin Zhao, Yujie Chen, Lixian Jiang, Xiaoyang Li, Bo Li, Yan Zhou

**Affiliations:** ^1^ Department of Radiology Renji Hospital School of Medicine Shanghai Jiao Tong University No. 160, Pujian Road, Pudong District Shanghai 200127 China; ^2^ Department of Radiology National Children's Medical Center Children's Hospital of Fudan University No. 399, Wanyuan Road, Minhang District Shanghai 201102 China; ^3^ Institute of Diagnostic and Interventional Radiology Shanghai Sixth People's Hospital School of Medicine Shanghai Jiao Tong University No. 600, Yishan Road, Xuhui District Shanghai 200233 China; ^4^ Department of Cardiology Zhongshan Hospital Fudan University No. 180, Fenglin Road, Xuhui District Shanghai 200025 China; ^5^ Morphology and Spatial Multi‐Omics Technology Platform Shanghai Institute of Nutrition and Health Chinese Academy of Sciences No. 320, Yueyang Road Shanghai 200031 China; ^6^ Department of Ultrasound in Medicine Shanghai Sixth People's Hospital School of Medicine Shanghai Jiao Tong University No. 600, Yishan Road, Xuhui District Shanghai 200233 China; ^7^ Department of Food Science and Technology School of Agriculture and Biology Shanghai Jiao Tong University No. 800, Dongchuan Road, Minhang District Shanghai 200240 China; ^8^ Key Laboratory of Anesthesiology (Shanghai Jiao Tong University) Ministry of Education No. 160, Pujian Road, Pudong District Shanghai 200127 China; ^9^ College of Health Science and Technology Shanghai Jiao Tong University School of Medicine No. 227, Chongqingnan Road Huangpu District Shanghai 200025 China

**Keywords:** extracellular vesicles, glycolysis, inflammatory and immune microenvironment, mTORC1‐HIF‐1α pathway, ulcerative colitis

## Abstract

Ulcerative colitis (UC) is a chronic inflammatory bowel disease mainly involving the colon and rectum, which features recurrent mucosal inflammation. The excessive production of reactive oxygen species (ROS) is a trigger for pathological changes such as cell apoptosis and disordered immune microenvironments, which are crucial for the progression of UC and can be a promising therapeutic target. Nowadays, the development of targeted therapeutic strategies for UC is still in its infancy. Thus, developing effective therapies based on ROS scavenging and elucidating their molecular pathways are urgently needed. Herein, a biomimetic nanoformulation (Pd@M) with cubic palladium (Pd) as the core and macrophage‐derived extracellular vesicles (MEVs) as the shell is synthesized for the treatment of UC. These Pd@M nanoformulations exhibit multienzyme‐like activities for effective ROS scavenging, excellent targeting ability as well as good biocompatibility. It is verified that Pd@M can regulate the polarization state of macrophages by inhibiting glycolysis, and decrease neutrophil infiltration and recruitment. In this way, the colonic inflammatory and immune microenvironment is remodeled, and apoptosis is prevented, ultimately improving colonic mucosal barrier function and alleviating colitis in the mouse model. This finding provides a promising alternative option for the treatment of UC patients.

## Graphical abstract

This study reports a novel macrophage‐derived extracellular vesicle‐coated palladium (Pd@M) nanoformulation for the targeted treatment of ulcerative colitis (UC). Pd@M can potently scavenge ROS, remodel the colonic inflammatory and immune microenvironments in vivo, and ultimately improve mucosal barrier function and induce the remission of UC, providing a promising alternative option for clinical application.

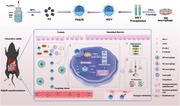

## 1 Introduction

As the most common inflammatory bowel disease worldwide, ulcerative colitis (UC) is characterized by mucus or mucus‐free bloody diarrhea. In particular, UC has a markedly disturbed inflammatory microenvironment including invasive pathogens, damaged tissues, and infiltrated immune cells during the active or remission stage, which is accompanied by overproduced interleukin (IL)−1β, IL‐6, tumor necrosis factor (TNF)‐α and other proinflammatory cytokines.^[^ ^1^, ^2^ ^]^ This nonspecific chronic inflammatory disease poses a significant challenge to the health care system as it not only manifests as colorectum mucosal inflammation but also triggers intestinal and extraintestinal complications that severely affect the life quality of patients. According to consensus‐based guidelines, systemic treatment relies heavily on the induction and maintenance of remission. Sulfasalazine and 5‐aminosalicylates are the first‐line treatments for UC with an expected remission rate of ≈50%, while glucocorticoids or immunosuppressive agents are indicated for patients who are refractory to 5‐aminosalicylates.^[^ ^3^, ^4^ ^]^ However, the results of clinical pharmacotherapy are unsatisfactory, as reflected by the high recurrence rate and a variety of complications.^[^ ^5^, ^6^ ^]^ Hence, exploiting more powerful and appropriate treatments for UC is imperative.

Reactive oxygen species (ROS) possess powerful oxidative capability and are detrimental to cells at high concentrations, but display complex signaling functions at low concentrations.^[^ ^7^ ^]^ Under the pathological conditions of UC, ROS oxidize intracellular proteins and lipid components, and damage deoxyribonucleic acid. It has been shown that ROS induces the transcription and translation of hypoxia‐inducible factor‐1α (HIF‐1α) through numerous regulators including the mammalian target of rapamycin 1 (mTOR1).^[^ ^8^ ^]^ By far, a number of nanoparticles (NPs), engineered bacteria, polymers, biomolecules, and organic molecules have been developed as potent mimic enzymes that exhibit catalase (CAT) or superoxide dismutase (SOD) activity, which can deplete excessive ROS.^[^ ^9^, ^10^, ^11^, ^12^ ^]^ In the biomedical field, nanomaterials can overcome the disadvantages of natural antioxidants such as short cycle time, increased toxic side effects from multiple dosing, and low absorption rate and bioavailability.^[^ ^13^ ^]^ Among them, noble metal NPs have attracted great interest due to their high stability, mass production, and tunable catalytic properties.^[^ ^14^ ^]^ Previously, palladium (Pd) has been reported to have intrinsic superior SOD and CAT‐like activities.^[^ ^15^, ^16^, ^17^ ^]^ However, existing studies have mainly focused on ROS scavenging and final manifestation, and it is still unclear exactly how Pd NPs further play a regulatory role beyond ROS. As a result, it is essential to uncover how Pd nanomedicine further modulates UC‐associated alternations in inflammatory and immune responses based on the scavenging capacity of ROS.

In addition to ROS, mucosal immune dysfunction is involved in the development of UC and the homeostasis of the intestinal microenvironment requires a counterbalance between innate and adaptive immune responses.^[^ ^18^ ^]^ Macrophages (Mφs), long‐lived cells of the innate immune system, play an important role in adaptive immunity, representing the first defense line of the body against pathogenic responses, and also get involved in antigen presentation and cytokine production. Furthermore, Mφs are essential for tissue homeostasis, which are roughly classified into two groups on the basis of their activation and microenvironment: M1 and M2.^[^ ^19^ ^]^ Both phenotypes are not stereotypical but change dynamically with the metabolism of Mφs, which thus leads to different roles in host defense against different pathogens, inflammation resolution, and wound healing.^[^ ^20^ ^]^ Previous studies have demonstrated that the mTOR1 and HIF‐1α are central regulators of the glucose metabolic switch in Mφs and the classical M1 polarization program is mainly up to glycolysis.^[^ ^21^, ^22^, ^23^, ^24^ ^]^ Specifically, mTOR1 senses cellular nutrients and regulates energy metabolism, which can modulate the function of innate and adaptive immune cell populations.^[^ ^25^ ^]^ In lipopolysaccharide (LPS)‐stimulated inflammatory Mφs, the activation of the mTOR1 pathway drives glycolytic metabolism by inducing HIF‐1α transcription. HIF‐1α binds to hypoxia‐responsive elements in promoters of target genes, like glycolytic enzymes and glucose transporter‐1 (GLUT1), to facilitate this glycolytic metabolic switch, which thereby regulates the polarization state of Mφs.^[^ ^21^, ^26^ ^]^ Then, activated M1 Mφs produce multiple cytokines, including TNF‐α, IL‐1β, IL‐6, inducible nitric oxide synthase (iNOS), etc., for the purpose of aggravating inflammation.^[^ ^19^ ^]^ Therefore, decreasing the polarization of glycolysis‐mediated M1 Mφs is a promising strategy for maintaining colonic homeostasis. However, a compromise between high catalyst activity, immune homeostasis, as well as targeting ability remains a challenging process in UC treatment.

**Scheme 1:**
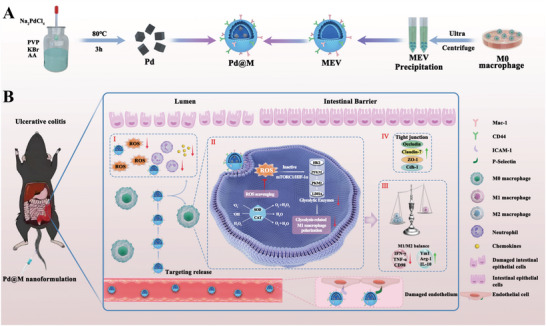
Schematic depiction of the synthesis, targeting ability, and mechanism of Pd@M in UC treatment.

Nanotherapeutics based on cell membranes have evolved into a useful approach to facilitating the targeting delivery of therapeutic agents as they integrate the functional diversity of nanomaterials with the biomimetic receptors and sensors of cell membranes.^[^ ^27^, ^28^, ^29^ ^]^ Specially, with the premise of ensuring optimal biocompatibility and targeting ability, macrophage‐derived extracellular vesicles (MEVs), have become a reliable alternative to macrophage membranes and can be used to develop biomimetic drug delivery systems targeting inflammatory regions because of their property to adhere to the inflamed endothelium of vascular tissue via particular ligands.^[^ ^30^, ^31^ ^]^ Here, MEVs‐coated cubic Pd (Pd@M), a biomimetic nanomedicine, was synthesized as an efficient ROS scavenger for the treatment of UC (**Scheme** **1**A). Pd@M possesses remarkable biocompatibility and multienzyme‐like activities, including CAT, SOD, and hydroxyl radical (‐OH) scavenging activities. Macrophage differentiation antigen 1 (Mac‐1) and cluster of differentiation 44 (CD44) on the MEV surface give Pd@M outstanding targeting ability since they can bind specifically to intercellular cell adhesion molecule‐1 (ICAM‐1) and P‐selectin, which are highly expressed on UC inflammation‐impaired endothelium. In in vitro and in vivo experiments, Pd@M improves the polarization status of Mφs by inhibiting the glycolytic pathway and blocks neutrophil infiltration and recruitment. Thus, the colonic inflammatory and immune microenvironment is reshaped, and cell apoptosis is prevented, which leads to improved colonic mucosal barrier function for the treatment of colitis (Scheme 1B). Importantly, it is found that Pd@M may regulate Mφ glycolysis levels through the inhibition of the mTORC1‐HIF‐1α pathway. This research provides a novel approach to the treatment of UC and advances the understanding of the mechanisms associated with Pd@M in regulating immune and inflammatory responses.

## 2 Results and Discussion

### 2.1 Synthesis and Characterization of Pd@M

**Figure 1:**
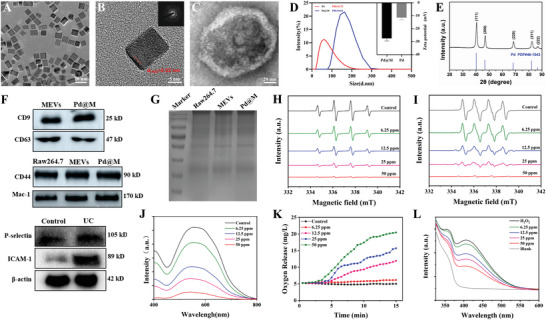
Construction, characterization, and ROS scavenging ability of Pd@M. A–C) TEM images of Pd, Pd@M. Scale bar: 20; 5; 20 nm. D) Size distribution and zeta potential of Pd and Pd@M. E) XRD spectrum of Pd. F) Western blot of CD9 and CD63 expression in MEVs and Pd@M (up). Western blot of CD44 and Mac‐1 in RAW264.7 membranes, MEVs, and Pd@M (middle). Western blot of P‐selectin and ICAM‐1 expression in the control group and UC group (down; *n* = 3). G) Analysis of protein compositions of macrophage membranes (RAW264.7), MEVs, and Pd@M by SDS‐PAGE. H) ESR curve of scavenging hydroxyl radical (·OH) of various groups with DMPO as the spin trap. I) ESR curve of scavenging superoxide anion (•O_2_^−^) of different groups with DMPO as the spin trap. J) Evaluation of the SOD‐like activities with distinctive concentrations of Pd@M by UV–vis utilizing a SOD kit. K) Oxygen concentration changes with different solutions at different time points. L) Evaluation of the CAT‐like activities with different groups by UV–vis utilizing a CAT kit.

To treat ulcerative colitis (UC), we first designed biomimetic nanoformulations that combine ROS scavenging and targeting properties. The palladium (Pd) nanocubes were optimally synthesized, whose morphology was described by transmission electron microscopy (TEM). As shown in standard TEM images (**Figure** **1**A,B), the vast majority of Pd NPs presented as relatively regular cubes with an average edge length of ≈15 nm. After their modification by macrophage‐derived extracellular vesicles (MEVs), the outer layer of Pd@M exhibited a membrane structure (Figure 1C). The dynamic laser scattering (DLS) result showed that the particle size increased from 51.89 nm to 168.5 nm and zeta potential decreased from −11.4 to −27.5 mV after MEVs encapsulation (Figure 1D). The structural properties and chemical composition of Pd were analyzed by performing X‐ray diffraction (XRD) and photoelectron spectroscopy (XPS), which revealed the face‐centered cubic structure of Pd nanocubes, with zero‐valent Pd being the dominant state (Figure 1E; Figure S1, Supporting Information). Moreover, the obtained MEVs were confirmed by TEM morphology, particle size distribution, and zeta potential (Figure S2, Supporting Information). Besides, the encapsulation efficiency of Pd@M was ≈71.3%, and the encapsulation number of individual MEVs was mainly three or four (Figure S3, Supporting Information). Pd@M NPs could be kept stable at 4 °C for more than 7 days (Figure S4A, Supporting Information) and also showed the Tyndall effect (Figure S4B, Supporting Information), indicating that Pd@M NPs are well dispersed in physiological environments. On the other hand, canonical markers CD9 and CD63 were verifiably expressed by Pd@M and MEVs (Figure 1F). The sodium dodecyl sulfate‐polyacrylamide gel electrophoresis (SDS‐PAGE) examination confirmed that RAW264.7 membranes, Pd@M, and MEVs were quite similar in protein composition (Figure 1G). This suggests that Pd@M has successfully and efficiently retained the MEV membrane proteins to take advantage of their bifunctionality. Previous reports show the adherence of macrophages (Mφs) to inflamed vascular pannus at the injury site owing to the high expression of adhesion molecules like P/E‐selectin and intercellular adhesion molecules (ICAMs).^[^ ^32^ ^]^ Subsequently, RAW264.7 membranes, Pd@M, and MEVs showed considerably abundant CD44 and Mac‐1, two crucial molecules during Mφ adhesion (Figure 1F). P‐selectin and ICAM‐1, whose ligands are matchable with CD44 and Mac‐1,^[^ ^33^ ^]^ were found to be highly expressed in the colonic tissues of UC model mice (Figure 1F). Overall, these results provide fundamental support for the targeting capability of Pd@M in UC treatment.

Usually found in various biological processes in the form of hydroxyl radicals (•OH), superoxide radicals (•O_2_^−^), or hydrogen peroxide (H_2_O_2_), reactive oxygen species (ROS) plays a critical role in intracellular functions and cellular signaling processes.^[^ ^34^, ^35^ ^]^ The excessive production of ROS disrupts the cellular redox balance and contributes to inflammatory conditions. The clearance of excess ROS and the restoration of a normal microenvironment are essential to alleviate inflammation. Therefore, the effects of Pd@M on typical ROS models (•OH, •O_2_^−^, and H_2_O_2_) were investigated. First, electron spin resonance (ESR) was utilized to assess the ability of Pd@M to remove •OH radicals. Fenton reaction was performed between Fe^2+^ and H_2_O_2_ to generate •OH radicals. The characteristic ESR spectrum for •OH/5‐dimethyl‐1‐pyrroline‐Noxide (DMPO) had a relative intensity of 1:2:2:1, and the peak intensity showed a markedly concentration‐dependent decrease with the addition of Pd@M (Figure 1H), which indicated that Pd@M can efficiently wipe out •OH radicals. Next, the xanthine/xanthine oxidase system was selected as the O_2_•^−^ generator. The ESR results showed a significant Pd@M concentration‐dependent decrease in the intensity of the characteristic 1:1:1:1 peak of •O2−/DMPO (Figure 1I). Similarly, the ultraviolet‐visible (UV–vis) analysis displayed the same conclusion (Figure 1J). Nitro blue tetrazolium (NBT) is oxidized by •O_2_^−^ to produce a blue high‐absorbance complex at 560 nm, while SOD‐like substances scavenge •O_2_^−^ and inhibit the formation of products. The increase in the concentration of Pd@M led to a gradual decrease in the absorbance at 560 nm, which further proves that Pd@M has outstanding SOD‐like activity, and serves as a catalyzer to decompose •O_2_^−^ into ordinary O_2_ and H_2_O_2_. Finally, O_2_ production was monitored with a dissolved oxygen meter to determine the CAT‐like activity of Pd@M nanocubes. It turned out that the capacity of O_2_ production increased with the increase of Pd@M concentration (Figure 1K). UV−vis spectrums observed similar results, which suggested that Pd@M reduced the absorbance at 415 nm (Figure 1L). Meanwhile, the enzyme kinetics result showed that the O_2_ production varied with the change of substrate H_2_O_2_ concentration over time, which was in accordance with the Michaelis–Menten kinetic equation. The K_m_ and V_max_ were 28.75 mM and 0.05 mg L^−1^ s^−1^, respectively (Figure S5A–C, Supporting Information). In addition, the efficiency of O_2_ production catalyzed by Pd@M was slightly higher in neutral than in acidic environments (Figure S5D, Supporting Information). The above data indicated that Pd@M nanocubes possess CAT‐like activity, namely catalyzing the conversion of H_2_O_2_ to O_2_ and H_2_O. More importantly, the ability of Pd@M to reduce ROS was similar to that of Pd, which proved that catalytic performance was little affected by the MEV modification (Figures S6 and S7, Supporting Information). In summary, Pd@M can act as an effective ROS scavenger and have the potential to promote the restoration of the inflammatory microenvironment.

### 2.2 Cell Endocytosis, ROS Scavenging Capacity, and Anti‐Apoptotic Ability of Biomimetic Pd@M In Vitro

**Figure 2:**
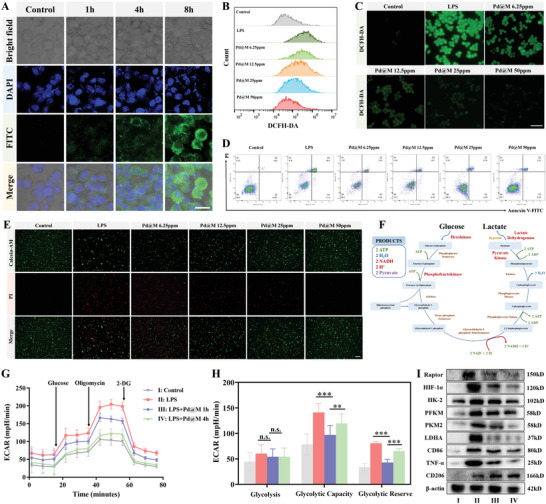
In vitro endocytosis, cytoprotective performance, and glycolysis regulation of Pd@M. A) Confocal microscopy images of RAW264.7 cells after 0, 1, 4, and 8 h of co‐incubation with FITC‐labeled Pd@M. Scale bar = 20 µm. B,C) Flow cytometry analysis and fluorescence microscopy images of RAW264.7 cells stained with DCFH‐DA after diverse treatments. Scale bar = 100 µm. D) Flow cytometry analysis of RAW264.7 cells receiving Annexin V‐FITC and PI staining after diverse treatments. E) Confocal microscopy images of RAW264.7 cells receiving Calcein AM/PI staining after diverse treatments. Scale bar = 50 µm. F) Schematic overview of the glycolytic pathway and important enzymes for evaluating glycolysis rate. G,H) ECAR reflected the glycolytic flux of RAW264.7 cells after varied treatments (*n* = 5). I) Representative western blot analyses showed the protein expression of Raptor, HIF‐1α, HK‐2, PFKM, PKM2, LDHA, CD86, TNF‐α, CD206, and β‐actin in RAW264.7 cells after varied treatments (*n* = 5). Data are displayed as mean ± standard deviation (SD). *p*‐values are calculated using a one‐way analysis of variance (ANOVA) with Tukey's post‐hoc test. ^**^ *p* < 0.01, ^***^ *p* < 0.001, n.s. implies no significant difference.

Nanomedicine engulfed by Mφs is crucial to delivering therapeutic benefits.^[^ ^27^ ^]^ Therefore, the uptake of Pd@M was investigated using RAW264.7 cells. RAW264.7 cells were used to incubate with fluorescein isothiocyanate (FITC)‐labeled Pd@M at different time periods (0, 1, 4, and 8 h), whose nuclei were stained by 2‐(4‐amidinophenyl)−6‐indolecarbamidine dihydrochloride (DAPI). Confocal images (**Figure** **2**A) and flow cytometry analysis (Figure S8, Supporting Information) demonstrated that the cellular uptake of Pd@M showed time dependence, which reached saturation after ≈8 h. These results confirm that Pd@M can be well endocytosed by RAW264.7 cells, which is a prerequisite for anti‐inflammatory therapy.

In view of the strong antioxidative capacity exhibited by Pd@M, their potential to scavenge ROS in vitro was evaluated. The stimulation of lipopolysaccharide (LPS) induced the inflammation of RAW264.7 cells^[^ ^36^ ^]^ that were stained by a fluorescent dye sensitive to ROS, namely 2′,7′‐dichlorofluorescin diacetate (DCFH‐DA). Flow cytometry analysis (Figure 2B) showed a marked right shift of the curve occurred in the LPS‐treated alone group. In contrast, the curve gradually shifted to the left when cells were pretreated with Pd@M. Fluorescence images (Figure 2C) showed similar results, indicating that Pd@M effectively removed intracellular ROS, thereby relieving the inflammatory response in vitro.

Subsequently, the cell viability of RAW264.7 cells was examined using the Cell Counting Kit‐8 (CCK8) assay to evaluate the therapeutic effect of Pd@M. Cells were first incubated with various concentrations (0–200 µg mL^−1^) of Pd@M for 24 and 48 h and exhibited no visible reduction in viability compared to the control group, which illustrated no apparent cytotoxicity to the cell line (Figure S9A, Supporting Information). Importantly, the cell death caused by LPS was gradually and significantly reversed with the increase in treatment concentration (Figure S9B, Supporting Information). The data also revealed that Pd@M‐ and Pd‐treated groups showed no significant difference (Figure S9C, Supporting Information). Meanwhile, cytoprotective capacity was also verified through flow cytometry sorting of Annexin‐V/PI staining, the result showed a dramatic increase in the proportion of viable cells and a decrease in the late apoptotic cells of the Pd@M‐50 µg mL^−1^ group (Figure 2D). The staining experiments of living/dead cells further confirmed this result. Calcein‐AM can distinguish living cells and excite green fluorescence, whereas PI is only capable of labeling dead cells and displaying red fluorescence. After being incubated with Pd@M at various concentrations, the red fluorescence gradually decreased with increasing Pd@M dose, and the Pd@M‐50 µg mL^−1^ group almost showed no significant red fluorescence compared with the LPS one, which indicated the dose‐dependent cytoprotective efficiency of Pd@M (Figure 2E). In addition to experiments with different concentrations, experiments were done with different nanocubes as well. Pd‐ and Pd@M‐treated groups showed no obvious difference in intracellular ROS level and cell apoptosis rate (Figures S10–S12, Supporting Information). Consequently, Pd@M can attenuate LPS‐induced cell apoptosis.

### 2.3 Regulation of Mφ Polarization by Inhibiting Glycolysis Metabolism

The glycolytic system not only functions as the first step in carbohydrate metabolism to produce adenosine triphosphate (ATP) that directly supplies the body with energy but also exerts a regulatory effect on the function of the immune system.^[^ ^26^ ^]^ In general, the uptake of glucose is realized by two transport modes: active glucose transport into the small intestinal epithelial cells of mammals through sodium‐dependent glucose transporters (SGLT) or facultative diffusion through glucose transporters (GLUTs) located in the membrane of diverse cells. Once entering the cells, glucose is oxidized via a stepwise mechanism.^[^ ^37^ ^]^ The first phase is glycolysis (see Figure 2F for exact flow) which occurs in the cytoplasm and is a process completed through the oxygen‐independent activity of 10 metabolic enzymes. The glycolytic rate is determined by rate‐limiting enzymes (hexokinase [HK] −2, phosphofructokinase‐muscle [PFKM] and pyruvate kinase M2 [PKM2]), which are regulated in an allosteric manner and important for regulating glycolytic flux.^[^ ^38^, ^39^, ^40^ ^]^ Glycolysis converts a single glucose molecule anaerobically to two pyruvate molecules and generates two ATP molecules. When oxygen is sufficient, the combined activities of glycolysis, the electron transport chain (ETC), and the tricarboxylic acid (TCA) cycle generally contribute to the generation of adequate ATP levels to meet the bioenergetic requirements of cells, tissues, and organisms to maintain steady‐state homeostasis.^[^ ^26^ ^]^ In the activated immune system, however, glycolysis can be rapidly induced despite being an inefficient means of producing ATP compared with O_2_‐dependent mitochondrial oxidative phosphorylation (OXPHOS), which offers rapid energy to meet the metabolic demands of activated immune cells.^[^ ^22^ ^]^

Various immune cells, including classically activated M1 Mφs, neutrophils, and dendritic cells, develop a high‐glycolysis phenotype once activated.^[^ ^41^, ^42^ ^]^ Glycolytic metabolism distinctly affects the function of immune cells and has a profound influence on the immune response. For example, Mφ activation may manifest as a classical/M1 phenotype or an alternative/M2 one, which displays crucial differences in metabolism. M1 polarization is featured by enhanced glycolysis along with reduced mitochondrial OXPHOS, while M2 features efficient OXPHOS.^[^ ^22^ ^]^ Glycolysis is essential to maintaining the infiltration capacity and microbicidal effect of M1 Mφs and enables them to initiate an immune response and produce ROS, NO, and pro‐inflammatory cytokines. By comparison, M2 Mφs upregulate arginase‐1 (Arg‐1), IL‐10, and other anti‐inflammatory cytokines, and release polyamines and growth factors, which thus promote angiogenesis and wound repair.^[^ ^43^ ^]^ Therefore, whether Pd@M could regulate the glycolysis of Mφs and affect Mφ polarization in an inflammatory state was assessed first. Seahorse technology was used to analyze metabolic flux. Extracellular acidification rate (ECAR), which indicates glycolytic rate, showed marked changes between the LPS‐stimulated group and the LPS+Pd@M‐treated group (Figure 2G,H), indicating that Pd@M can downregulate Mφ glycolysis in a treatment time‐dependent manner. Similar results were also found in oxygen consumption rate (OCR), i.e., Pd@M had significantly improved basal respiration OCR, maximal respiration OCR, and ATP production, suggesting that Pd@M can reverse LPS‐induced downregulation of mitochondrial phosphorylation function in Raw264.7 cells (Figure S13, Supporting Information). Correspondingly, the LPS‐treated group showed a significant increase in the protein contents of key glycolytic enzymes such as HK‐2, PFKM, PKM2, and lactic dehydrogenase A (LDHA) compared with the control one, but a progressive decrease with the increasing time of Pd@M treatment (Figure 2I; Figure S14, Supporting Information). Also, the obtained data showed that the LPS+Pd@M group exhibited significantly lower protein levels of M1 polarization markers (CD86 and TNF‐α) than the LPS group, while the M2 marker (CD206) showed an increasing trend after diverse interventions (Figure 2I; Figure S14, Supporting Information). These results verify that Pd@M nanocubes can reduce M1 Mφ polarization and its associated inflammation by inhibiting Mφ glycolysis.

Furthermore, high‐flux glycolysis in activated immune cells is regulated by the mammalian target of rapamycin (mTOR1) and hypoxia‐inducible factor‐1α (HIF‐1α) with a wide range of downstream targets to facilitate this process.^[^ ^25^ ^]^ The mTORC1 pathway integrates no less than five main intracellular and extracellular signals‐ hypoxia, stress, energy status, growth factors, and amino acids, and translates them into suitable biological processes, including energy metabolism, protein and lipid synthesis, and autophagy.^[^ ^44^ ^]^ In the innate immune system, the mTORC1 network can be activated by a variety of extracellular ligands like LPS and Toll‐like receptor (TLR) ligands. The activation of the mTOR1 pathway drives glycolytic metabolism by activating the transcription and translation of HIF‐1α. HIF‐1α, a heterodimeric protein comprising an oxygen‐sensitive α subunit, is usually stable under anaerobic/hypoxic conditions and is responsible for the regulation of glycolysis in hypoxia. It can accumulate within the cytoplasm and subsequently translocate to the nucleus to form HIF heterodimers.^[^ ^26^, ^45^ ^]^ Upon binding to hypoxia‐responsive elements (HREs) within the promoter/enhancer regions of target gene, these heterodimers initiate gene transcription and stimulate an array of adaptive responses to hypoxia, including the reprogramming of HIF‐1α‐dependent glucose metabolism to reduce dependence on O_2_‐dependent energy production. To be specific, superfluous HIF‐1α upregulates the expression of genes encoding GLUTs (like GLUT1 and GLUT3) and glycolytic pathway enzymes, including HK‐2, PFKM, PKM2, and LDHA, and suppresses OXPHOS to achieve an adaptive increase in glycolytic flux.^[^ ^46^, ^47^, ^48^ ^]^ Notably, the mTORC1 (Raptor) and HIF‐1α protein levels in the Pd@M‐treated group showed a significant decrease compared with those in the LPS group, consistent with the trend of proteins related to the glycolytic pathway (Figure 2I; Figure S14, Supporting Information). Taken together, Pd@M may inhibit M1‐phenotype Mφ polarization by blocking mTORC1‐HIF‐1α‐mediated glycolysis, thus exerting its anti‐inflammatory and anti‐apoptotic effects.

### 2.4 Biodistribution and Targeting Accumulation of Pd@M In Vivo

**Figure 3:**
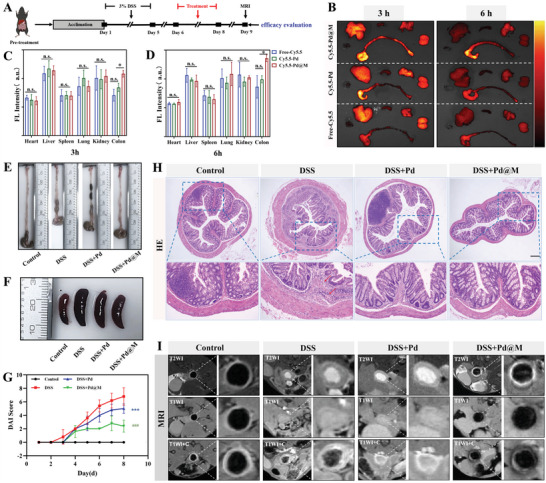
Superior targeting ability and favorable therapeutic efficacy of Pd@M in the DSS‐induced colitis mice model. A) Schematic overview of the experimental design of the DSS‐induced UC model mice. B) Fluorescence imaging of the main organs (hearts, livers, spleens, lungs, kidneys, and colons) of colitis mice after the intravenous administration of free‐Cy5.5, Cy5.5‐Pd and Cy5.5‐Pd@M for 3 and 6 h (*n* = 3). C,D) Statistics of fluorescence intensity of main organs at 3 and 6 h (*n* = 3). E–G) Typical macroscopic appearance of colons and spleens and the DAI score of mice with the indicated treatments of each group on day 9 (*n* = 5). H) Representative H&E images of colon tissues from each group on day 9 (*n* = 5). Scale bar = 100 µm. I) Representative MRI images of colon tissues from each group on day 9 (*n* = 5). Data are demonstrated as mean ± SD. *p*‐values are calculated using one‐way ANOVA with Tukey's post‐hoc test. *p < 0.05, ^***^ *p* < 0.001, n.s. implies no significant difference.

UC is related to a series of inflammatory cascades, which thus makes inflammation‐targeted therapy an emerging concept to guide UC treatment over the past few years.^[^ ^49^ ^]^ Therefore, the inflammation targeting ability and in vivo biodistribution of biomimetic Pd@M nanocubes were examined after the intravenous injection of free Cy5.5, Cy5.5‐labeled Pd, or Cy5.5‐labeled Pd@M into mice with colitis. Major organs and colons were collected for ex vivo imaging at 3 and 6 h after injection. It was observed that Cy5.5 in all formulations accumulated mainly in the liver, probably because of its non‐specific uptake by the mononuclear phagocyte system of the liver.^[^ ^50^ ^]^ Of note, Cy5.5‐labeled nanocubes were significantly different only in colons rather than in other organs (**Figure** **3**B–D). It was obvious that the colonic fluorescence was strongest for Cy5.5‐labeled Pd@M, followed by Cy5.5‐labeled Pd and free Cy5.5. In addition, the Cy5.5‐Pd@M group exhibited more fluorescence expression and significantly greater fluorescence intensity compared with the Pd group at the two‐time points. Pd@M maintains an efficient targeting capacity in the impaired colon for at least 6 h. In general, the above results demonstrate the superior inflammation‐targeting ability of Pd@M, which thus enhances the therapeutic effects of its payload.

### 2.5 Amelioration of DSS‐Induced Colitis

Based on the outstanding anti‐inflammatory and anti‐apoptotic activities and the excellent targeting ability of Pd@M nanocubes in vitro, a colitis mouse model induced by dextran sulfate sodium (DSS) was used for assessing whether Pd@M has therapeutic effects in vivo (Schematically shown in Figure 3A). Mice were classified into four groups at random: control, model (DSS), DSS+Pd, and DSS+Pd@M groups. The Pd@M‐treated group showed a significantly longer colon length, lighter spleen weight, lower disease activity index (DAI) score, and body weight compared with the DSS group (Figure 3E–G; Figure S15, Supporting Information). In addition, we used MEVs treated with the same polycarbonate membrane filtration operations for the DSS‐induced colitis model. As a result, no significant changes in colon lengths, body weights, and DAI scores were found between the DSS+MEVs group and the DSS group (Figure S16, Supporting Information). In hematoxylin‐eosin (HE) staining analysis, mice that suffered from colitis showed a decrease in the goblet and superficial epithelial cells, accompanied by inflammatory cell infiltration into the mucosa and submucosa (Figure 3H). Besides, the DSS group showed irregular thickening and inhomogeneous enhancement of the colonic wall with mucosal edema in magnetic resonance imaging (MRI) images (Figure 3I). As expected, these lesions were remitted profoundly after Pd or Pd@M treatment, with Pd@M possessing a conspicuously better treatment effect (Figure 3H,I). In conclusion, these results confirm that Pd@M has great therapeutic effects on DSS‐induced colitis mice.

### 2.6 Improvement of the Colonic Immune and Inflammatory Microenvironment by Pd@M

**Figure 4:**
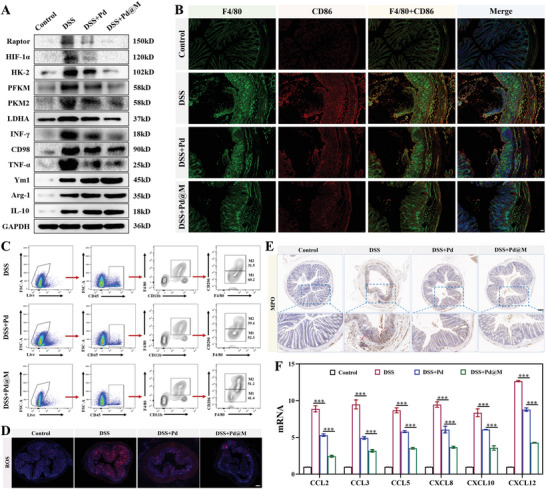
Inhibition of glycolysis‐associated M1 Mφ polarization and improvement of the inflammatory microenvironment by Pd@M. A) Western blot analysis of the expression of Raptor, HIF‐1α, HK‐2, PFKM, PKM2, LDHA, INF‐γ, CD98, TNF‐α, Ym1, Arg‐1, IL‐10, and GAPDH in mice colons from four groups on day 9 (*n* = 5). B) Immunofluorescent staining of F4/80, CD86, and DAPI of colon tissues from each group on day 9 (*n* = 5). Scale bar = 50 µm. C) Gating strategy for CD45^+^CD11b^+^F4/80^+^CD206^+^ and CD45^+^CD11b^+^F4/80^+^CD206^−^ Mφs in mice from three groups on day 9 (*n* = 5). D) Representative images of DHE staining for detecting ROS production in the colon tissues of each group on day 9 (*n* = 5). Scale bar = 200 µm. E) Representative MPO staining of colon tissues from each group on day 9 (*n* = 5). Scale bar = 200 µm. F) Relative mRNA levels of CCL2, CCL3, CCL5, CXCL8, CXCL10, and CXCL12 in colon tissues from each group on day 9. Data are shown as mean ± SD. *p*‐values are calculated using one‐way ANOVA with Tukey's post‐hoc test. ^***^ *p* < 0.001.

The effects of inhibiting high‐glycolysis immune cell phenotypes have been studied in the in vivo models of systemic lupus erythematosus, experimental autoimmune neuritis, and other autoimmune conditions. The results indicated that glycolysis inhibition could have a profound influence on innate and adaptive immune responses with significant remission in clinical signs.^[^ ^51^, ^52^ ^]^ Given that Pd@M can reduce the glycolysis mediated by mTORC1/HIF‐1α and associated M1 Mφ polarization in vitro, it was investigated whether Pd@M could successfully interfere with the glycolytic pathway in vivo. According to the Western blot results, the expression of Raptor (mTORC1) and HIF‐1α proteins, as well as glycolytic enzymes (HK‐2, PFKM, PKM2, and LDHA) was significantly decreased in the DSS+Pd@M group compared with the DSS group (**Figure** **4**A; Figure S17, Supporting Information). Colon tissue ATP assay also showed that Pd@M treatment remarkedly reversed the decline of ATP production in DSS mice (Figure S18, Supporting Information). The above indicated that the glycolysis level in the inflamed colonic tissues was diminished, consistent with the previous metabolic alternations of Mφs in vitro. Similar to the results of in vitro experiments, immunofluorescence analysis revealed that the NP‐treated groups showed a reduction in CD86 expression in comparison with the DSS group, with Pd@M therapy having the most pronounced effect (Figure 4B). Specifically, we observed a marked increase in CD45^+^CD11b^+^F4/80^+^CD206^+^ Mφs and a significant decrease in CD45^+^CD11b^+^F4/80^+^CD206^−^ Mφs in the Pd@M treated group compared to the DSS group (Figure 4C). In addition, TNF‐α and INF‐γ (relating to M1 Mφ polarization) were dramatically decreased, whereas anti‐inflammatory cytokines (Ym1, Arg‐1, and IL‐10 representing M2 Mφ polarization) were significantly increased after Pd@M treatment (Figure 4A; Figure S17, Supporting Information). These results suggest that Pd@M may promote a shift from M1 to M2 Mφ polarization in DSS mice, in which glycolysis inhibition may play a pivotal role. Moreover, CD98 is a type II transmembrane glycoprotein that shows the highest expression in the gastrointestinal tract and tubules of the kidney. INF‐γ‐mediated CD98 upregulation aggravates intestinal inflammation by controlling immune cell activation, the host response to intestinal flora, and intestinal epithelial reconstitution.^[^ ^53^, ^54^ ^]^ The data of this study also showed that Pd@M treatment suppressed CD98 expression to a large extent (Figure 4A; Figure S17, Supporting Information), which again indicated the relief of inflammation. Then, dihydroethidium (DHE) staining was used to detect the ROS level in the colonic mucosa with the aim of further validating the in vivo therapeutic mechanism of Pd@M. The DSS group exhibited enormous red fluorescence, which was positively associated with ROS content. However, the ROS level was significantly lower in the DSS+Pd@M group, demonstrating that Pd@M has the capacity to act as a competent antioxidant to scavenge ROS in vivo (Figure 4D). During the process of UC, neutrophils are one of the first cell types to be recruited to tissues, and subsequent neutrophil activation leads to the release of chemical mediators that in turn get involved in recruiting other immune cells to exacerbate inflammation.^[^ ^55^ ^]^ Finally, the excessive recruitment and accumulation of neutrophils cause damage to the mucosa and increase the permeability of the intestinal tract to bacteria. Myeloperoxidase (MPO, a biomarker of neutrophil infiltration)‐positive neutrophils were markedly recruited in the DSS group as expected, but decreased sequentially after Pd and Pd@M treatments (Figure 4E). Inflammatory chemokines such as C‐C motif chemokines 2, 3, and 5 (CCL2, CCL3, and CCL5) and C‐X‐C motif chemokine ligands 8, 10 and 12 (CXCL8, CXCL10, and CXCL12) are major mediators of inflammatory cells homing to the injured/inflammatory tissues.^[^ ^56^ ^]^ Similarly, our findings revealed that Pd@M treatment dramatically decreased the mRNA levels of chemokines, which thus potentially reduced the infiltration of inflammatory cells (Figure 4F). To sum up, Pd@M can improve the colonic immune and inflammatory microenvironment by reducing glycolysis‐related M1 Mφ polarization and inflammatory cell infiltration, which therefore attenuates the progression of UC.

### 2.7 Restoration of Colonic Mucosa Barrier Function by Pd@M

**Figure 5:**
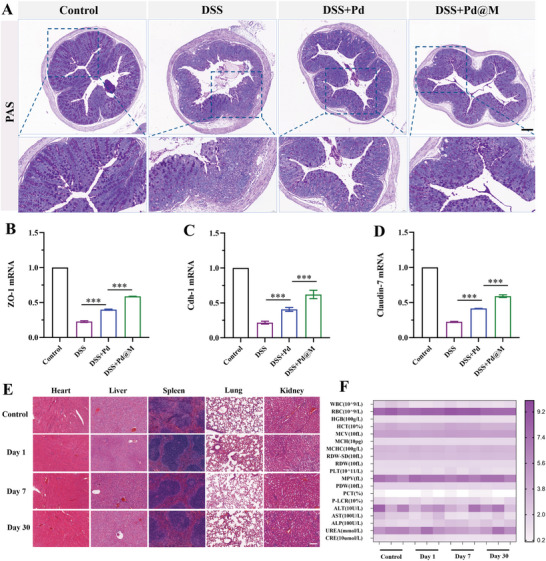
Improvement of intestinal mucosal barrier function by Pd@M. A) Representative images of PAS staining of colon tissues from each group on day 9 (*n* = 5). Scale bar = 200 µm. B–D) Relative mRNA levels of ZO‐1, Cdh‐1, and Claudin‐7 of colon tissues from each group on day 9 (*n* = 3). Data are shown as mean ± SD. *p*‐values are calculated using one‐way ANOVA with Tukey's post‐hoc test. ^***^ *p* < 0.001. E,F) Biocompatibility assessment of Pd@M (*n* = 5). H&E staining of the main organs (hearts, livers, spleens, lungs, and kidneys) and biochemical assays of mice receiving Pd@M via intravenous administration (days 1, 7, and 30).

The epithelial barrier covered by the mucosal layer not only provides a mechanical barrier between host immune cells and luminal microorganisms but also synthesizes antimicrobial peptides, making it the first line of defense of the mucosal immune system. UC features the activation of the mucosal immune system, accompanied by reduced synthesis and altered sulphation of colonic mucin subtypes (mucin 2), extensive epithelial cell apoptosis, and the reduced complexity of tight junctions (TJs) between epithelial cells, which ultimately impairs the function of the epithelial barrier and destroys tissues.^[^ ^57^ ^]^ Based on the improved immune and inflammatory microenvironment in vivo, the influence of Pd@M on the barrier function of the colonic mucosa was evaluated. Periodic acid Schiff (PAS) staining showed that goblet cells in colon tissues displayed the function of secreting mucin to protect the intestinal mucosa. It turned out that Pd@M vigorously reversed the DSS‐induced decrease in colonic goblet cells (**Figure** **5**A). Furthermore, representative TJ‐associated molecules, which are of importance for maintaining the barrier integrity of colonic mucosa, were assessed by quantitative polymerase chain reaction (qPCR). The DSS group showed a significantly lower mRNA expression of Zona occludens 1(ZO‐1), Occludin, and Claudin‐1 than the control group. In contrast, Pd@M treatment abated the decline of the mRNA expression of TJ‐associated molecules (Figure 5B–D). Taken together, Pd@M can effectively protect the mucosa from inflammation‐induced damage.

### 2.8 Biocompatibility of Pd@M

Biosafety assessment was performed by administering a single dose of Pd@M (10 mg kg^−1^) intravenously to wild‐type mice. On days 1, 7, and 30 after injection, no distinguishable hemorrhage, necrosis, inflammatory lesions, or tissue injuries were observed in the major organs of the mice that received Pd@M management (Figure 5E). Meanwhile, the biochemical analysis and blood routine parameters of the Pd@M group were not significantly different from those of the control group (Figure 5F), which demonstrated the excellent biocompatibility of Pd@M in vivo.

## 3 Conclusion

In summary, we have constructed biomimetic Pd@M nanoformulations with strong multiple enzyme activities, excellent targeting ability, and remodeling function of immune homeostasis. Specifically, the Pd@M nanosystem mimics the activities of multiple enzymes (SOD, CAT, and eliminating •OH) to catalyze the cascade reactions of ROS scavenging. Mac‐1 and CD44 of the MEVs shell have high affinity for ICAM‐1 and P‐selectin, which are highly expressed on UC inflammation‐impaired endothelial cells, endowing Pd@M with targeting ability. Therefore, these Pd@M NPs could be guaranteed to accumulate and exert therapeutic effects in the lesion of UC. In vitro and *vivo* experiments demonstrated that the Pd@M nanoformulations can regulate the polarization status of Mφs by inhibiting the mTORC1‐HIF‐1α‐mediated glycolytic pathway, i.e., decreasing M1 polarization and increasing M2 polarization, which is accompanied by the reduction of neutrophil infiltration and recruitment. Thus, the remodeling of the colonic inflammatory and immune microenvironment is promoted, and the barrier function of colonic mucosal gets improved, which effectively alleviates colitis. This work sheds light on the multiple regulatory pathways of Pd@M and provides a potential alternative strategy for their future clinical applications.

## 4 Experimental Section

### Ethical Statement

Animal care and experimental procedures were approved by the Institutional Animal Care and Use Committee of Shanghai Rat and Mouse Biotech Co. (No. 202210[13]).

### Statistical Analysis

Data analysis was conducted using SPSS software version 25.0 (IBM Co., Armonk, NY). Data normality was determined using the Shapiro‐Wilk test. One‐way analysis of variance (ANOVA) testing followed by Tukey's post‐hoc test was used to compare the differences in groups. Data were presented as mean ± standard deviation (SD) from at least three independent repeats. *p* < 0.05 was considered statistically significant. Graphs were plotted using GraphPad Prism (version: 9.0.0; GraphPad Prism Software Inc, San Diego, CA) and OriginPro (version: b9.5.1.195; OriginLab Co., Northampton, MA).

## Conflict of Interest

The authors declare no conflict of interest.

## Supporting information

Supporting Information

## Data Availability

The data that support the findings of this study are available from the corresponding author upon reasonable request.
