# Supplementary material for: Macrophage‐Derived Extracellular Vesicles‐Coated Palladium Nanoformulations Modulate Inflammatory and Immune Homeostasis for Targeting Therapy of Ulcerative Colitis
Source: Adv Sci (Weinh). 2023 Oct 9;10(33):2304002. doi: 10.1002/advs.202304002 (PMC10667822; doi:10.1002/advs.202304002)
Supplement: Supplementary file 1 — Supporting Information [file ADVS-10-2304002-s001.pdf]

ADVANCED SCIENCE
Open Access

Supporting Information

for *Adv. Sci.*, DOI 10.1002/advs.202304002

Macrophage-Derived Extracellular Vesicles-Coated Palladium Nanoformulations Modulate Inflammatory and Immune Homeostasis for Targeting Therapy of Ulcerative Colitis

Jiahui Cheng, Yiming Zhang, Liang Ma, Wenxian Du, Qiang Zhang, Rifeng Gao, Xinxin Zhao, Yujie Chen, Lixian Jiang, Xiaoyang Li, Bo Li* and Yan Zhou**

Supporting Information

Macrophage-derived Extracellular Vesicles-coated Palladium Nanoformulations Modulate Inflammatory and Immune Homeostasis for Targeting Therapy of Ulcerative Colitis

Jiahui Cheng[1, a], Yiming Zhang[1, a], Liang Ma[1, b], Wenxian Du[c], Qiang Zhang[c], Rifeng Gao[d], Xinxin Zhao[a], Yujie Chen[e], Lixian Jiang[f], Xiaoyang Li[*, g], Bo Li[*, a, h], Yan Zhou[*, a]

a. Department of Radiology, Renji Hospital, School of Medicine, Shanghai Jiao Tong University; No. 160, Pujian Road, Pudong District, Shanghai 200127, China
b. Department of Radiology, Children's Hospital of Fudan University, National Children's Medical Center; No. 399, Wanyuan Road, Minhang District, Shanghai 201102, China
c. Institute of Diagnostic and Interventional Radiology, Shanghai Sixth People's Hospital, School of Medicine, Shanghai Jiao Tong University; No. 600, Yishan Road, Xuhui District, Shanghai 200233, China
d. Department of Cardiology, Zhongshan Hospital, Fudan University; No. 180, Fenglin Road, Xuhui District, Shanghai 200025, China
e. Morphology and spatial multi-omics technology platform, Shanghai Institute of Nutrition and Health, Chinese Academy of Sciences; No. 320, Yueyang Road, 200031, Shanghai, China
f. Department of Ultrasound in Medicine, Shanghai Sixth People's Hospital, School of Medicine, Shanghai Jiao Tong University; No. 600, Yishan Road, Xuhui District, Shanghai 200233, China
g. Department of Food Science and Technology, School of Agriculture and Biology, Shanghai Jiao Tong University; No. 800, Dongchuan Road, Minhang District, Shanghai 200240, China
h. Key Laboratory of Anesthesiology (Shanghai Jiao Tong University), Ministry of Education; No. 160, Pujian Road, Pudong District, Shanghai 200127, China

Jiahui Cheng, Yiming Zhang and Liang Ma are first co-authors.

*Corresponding Author:** Xiaoyang Li, Bo Li and Yan Zhou.

Experimental Section

Chemicals and Materials: Sodium tetrachloropalladate (Na_2PdCl_4), L-ascorbic acid (AA), citric acid (CA), poly (vinyl pyrrolidone) (PVP, MW ≈ 55,000), and potassium bromide (KBr) were purchased from Sigma-Aldrich and used as received without further purification. Deionized (DI) water with a resistivity of 18.2 M cm was used to prepare all aqueous solutions.

Characterizations: TECNAI G2 high-resolution transmission electron microscope operating at 200 kV was used to obtain transmission electron microscopy (TEM) images. Mastersizer 3000 nano particle analyzer (Malvern, UK) was performed to measure dynamic light scattering (DLS). The X-ray diffraction patterns of the products were collected on Rigaku-D/max 2500 V X-ray diffractometer (Bruker, Germany) using Cu-Kα radiation (λ = 1.5418 Å) with the operation voltage and current kept at 40 kV and 40 mA. Shimadzu UV-2501 spectrophotometer recorded UV-Vis absorption spectrum. The concentration of Pd was quantified using inductively coupled plasma optical emission spectrometry (ICP-OES). Confocal laser scanning microscopy (Zeiss LSM 780, Carl Zeiss Inc., Jena, Germany) was used for acquiring fluorescence images.

Synthesis of 18 nm Pd Cubes: Different sizes of Pd cubes are synthesized using the method reported by Xia's group [1]. In general, to synthesize 18 nm Pd cubes, 60 mg ascorbic acid, 105 mg PVP (MW ≈ 55,000), 600 mg KBr are mixed together in 8 mL water and then heated at 80 °C for 10 min, followed by a rapid injection of 3.029 mL Na_2PdCl_4 aqueous solution (0.0640 M) into the reaction solution. After 3 hours, the products are collected by centrifugation with the help of acetone and re-dispersed in 20 mL of water for further use.

Synthesis of Pd@M Biomimetic Nanoformulations: After stimulation of macrophages (RAW264.7) with cytochalasin B (10 μg/ml) for 2 hours, the supernatant was collected and centrifuged at 500 g for 10 minutes; 3000 g for 10min；10000 g for 30min, and finally the supernatant was collected. Centrifuge 100000 g for 16h at 4 °C to enrich the exocrine body and resuspend with normal saline for further use. The obtained macrophage-derived extracellular vesicles (MEVs) were mixed with Pd at the mass ratio of 1:5 and sonicated in ice water bath for 3 minutes at 60W. To remove the agglomerated particles, the mixed solution after ultrasound was filtered through 800nm and 200nm polycarbonate membrane respectively. After negative staining with *uranyl acetate* or *tungsten phosphate*, the morphology of MEVs and Pd@M was verified by TEM. The size distribution of

MEVs was analyzed by nanoparticle tracking analysis (NTA; NanoSight NS300, Malvern Panalytical co., Malvern, UK), and zeta potential of MEVs were also confirmed. The encapsulation efficiency of Pd@M was analyzed by randomly selecting ten fields of view, and the percentage of number of Pd nanocubes encapsulated in a single MEV was analyzed and calculated by randomly selecting one hundred Pd@M NPs. Furthermore, CD9 and CD63, the specific expression of two markers on MEVs and Pd@M were validated by western blot. According to a previous article, sodium dodecyl sulfate-polyacrylamide gel electrophoresis (SDS-PAGE) was performed to investigate the protein compositions of the macrophages (RAW264.7), MEVs and Pd@M [2]. Meanwhile, we examined the expression of the relevant target protein molecules including CD44 and Mac-1 in RAW264.7 membranes, MEVs and Pd@M, as well as the expression of their corresponding ligands P-selectin and ICAM-1 in colonic tissues before or after inflammatory stimulation by western blot.

Determination of ROS Scavenging Capability: ESR spectroscopy was used to investigate the •OH scavenging ability of Pd@M biomimetic nanoformulations. In a typical determination, DMPO (5 μL 10 M) as a trapping agent was dissolved in PBS buffer (45 μ 50. 10 mM), followed by the addition of Fe^{2+} (20 μ L 50mM) to catalyzes the production of hydroxyl radical (·OH) from H_2O_2. The amount of •OH was quantified by intensity of the ESR amplitude in the absence and with the addition of different components of nanozyme.

The $•O_2^-$ scavenging ability was explored by ESR spectroscopy. Under ultrasonic treatment, 70 μg of KO_2 was added to 200 μL of the 18-crown-6 in DMSO solution (0.7 mM) to generate $•O_2^-$ and followed by the addition of DMPO (50 μL) as a trapping agent. Then the ESR signal was collected for quantitatively estimate the $•O_2^-$ scavenging ability without and with the addition of different components of nanozymes. Furthermore, the Superoxide Dismutase (SOD) Activity Assay Kit (Beijing Boxbio Science & Technology Co., Ltd) was also used to determine SOD-like activity of Pd@M. The reaction system of xanthine and xanthine oxidase can produce superoxide anion, O_2-reducible Nitrogen blue tetrazolium generates blue formazan, which has a characteristic absorption peak at 560 nm; SOD can scavenge $•O_2^-$ and thus reduce the production of formazan, so the lighter color of the reaction solution indicated the higher SOD activity. The change in absorbance at 560 nm spectrum can characterize the activity of SOD.

Spectra Hydrogen peroxide content detection kit (Beijing Solarbio Science & Technology Co., Ltd) was used to evaluate the CAT activity of Pd@M biomimetic nanoformulations. The CAT activity of the nano-enzyme removes peroxides, and the remaining peroxides oxidize $TiSO_4$ to form yellow titanium persulfate, which is absorbed at 415nm in UV−vis. As the absorbance decreased, the CAT activity of the nanozyme increased. Furthermore, the dissolved oxygen meter (Leici Co., Ltd.) was also used to assess the CAT ability of Pd@M. The higher the oxygen production rate or the final oxygen production, indicated the higher CAT like enzyme activity of the nano-enzymes. The kinetic analysis of CAT-like enzyme activity for Pd@M NPs was calculated based on the results of oxygen generation [3]. The dissolved oxygen profile of the reaction system was recorded as a function of substrate concentration and time. The enzyme kinetic parameters were acquired using the following Michaelis-Menten equation: $V = V_{max}[S]/(K_m+[S])$. In the Michaelis–Menten equation, V_{max} is the reaction rate at which Pd@M NPs is saturated with substrate H_2O_2. [S] is the concentration of substrate H_2O_2. The K_m value is called the Michaelis-Menten constant.

Cellular Uptake: RAW264.7 cells (Institute for Biological Sciences, Shanghai, China) were seeded at 2×10^5 cells per well into 6-well plates overnight, and the cells were stimulated by LPS (200 ng/ml) for 24 h and then incubated with FITC labeled Pd@M (50 μg/mL) for 1, 4 and 8 h. The cell uptake of Pd@M was tested by laser confocal microscopy (LSM 880 Basic Operation, Zesiss, *Oberkochen*, Germany) and flow cytometry (BD Bioscience, Brea, CA, USA). The data of flow cytometry was analyzed using FlowJo (FlowJo 10.8.1).

Cytotoxicity and Cell Viability: RAW264.7 cells were seeded at 1×10^4 cells per well in 96-well plates overnight. Cytotoxicity and viability of RAW264.7 cells were measured using CCK-8 assay (Beyotime Biotechnology, Shanghai, China). Briefly, to evaluate cytotoxicity, adherent cells were then co-cultured with different concentrations of Pd@M (0-200 μg/mL) for 24 and 48 h, respectively. Similarly, to perform cell viability assay, adherent cells were then divided into six groups, which given glucose-free medium containing LPS (1000 ng/ml) for 4 h, and the control group was replaced with normal medium. Then the culture mediums were replaced with different concentrations of Pd@M (6.25, 12.5, 25 and 50 μg/mL) and co-incubated for 4 h. CCK-8 kit (Beyotime Biotechnology, Shanghai, China) was used to detect cell viability after co-incubation for 2 h, live cells that react with the reagent can be detected by a microplate reader.

Anti-ROS and Anti-apoptotic Effects of Pd@M in Vitro: RAW264.7 cells were incubated in 6-well

plates overnight. After LPS (200 ng/ml) pretreated for 24 h, different concentrations of Pd@M (6.25, 12.5, 25 and 50 μg/mL) were added and incubation for 4 h, then stained with Calcein-AM/PI (Beyotime Biotechnology, Shanghai, China) and DCFH-DA (Beyotime Biotechnology, Shanghai, China), respectively. Fluorescence microscope (Leica Microsystems CMS GmbH, Wetzlar, Germany) were used to obtain fluorescent images of diverse groups. In addition, the proportions of live and dead cells (stained with Annexin-X and PI; Beyotime Biotechnology, Shanghai, China), and intracellular ROS levels (stained with DCFH-DA; Beyotime Biotechnology, Shanghai, China) were also quantified by flow cytometry (BD Bioscience) according to the manufacturer's instructions.

Seahorse Analysis: The extracellular acidification rate (ECAR) was measured by Seahorse XF Glycolysis Stress Test Kit [4]. 1×10^4 cells were collected into the Seahorse XF Flux Pak culture microplate for 24 h and centrifuged after 4 h of treatment with various materials. Seahorse XF24 Analyzer (Agilent, USA) were used to detect the real-time ECAR according to the instruction manual.

Induction of Colitis and Treatment Protocol: Male C57BL/6 mice (6–8 weeks old, 20 to 25 g; SLRC Laboratory Animal, Shanghai, China) were placed in groups of five mice per cage and acclimatized for 1 week before inclusion in the study. Mice were fed with 3% (w/v) DSS (molecular weight: 36,000 to 50,000, Yeasen Biotechnology Shanghai Co., Ltd) for 5 consecutive days to induce colitis [5]. To confirm whether synthetic NPs have an effect on colitis, the mice were randomly divided into four groups: control group, DSS group, DSS+Pd group, DSS+Pd@M group. The mice with DSS-induced colitis were intravenously injected with different NPs (0.5 mg/kg) for 3 consecutive days (days 6, 7 and 8), whereas the control group drank the same volume of distilled water and injected normal saline.

In Vivo Distribution and Inflammation-targeting Ability: Normal and colitis mice were administered with Cy5.5-labeled NPs (0.5 mg/kg). Wild-type mice injected with an equal amount of Cy5.5 were set as controls. After 3 and 6 h, mice were sacrificed, the main organs and colons were collected. In Vivo Elite imaging system (Vieworks Co., Ltd, Korea) was used to observe and quantify the fluorescence distribution of NPs in the colon, heart, liver, spleen, lung, and kidney.

Symptoms Evaluations: During the 8 days induction and treatment of colitis, the body weight, stool consistency and rectal bleeding of mice were observed and recorded every day. The disease activity

index (DAI) scores were calculated using the following indexes: (a) body weight loss: 0 points = none; 1 points = 1-5%; 2 points = 5-10%; 3 points = 10-15%; 4 points = over 15%; (b) stool consistency: 0 points = normal; 2 points = loose stools; 4 points = diarrhea; (c) gross bleeding: 0 points = normal; 2 points =hemoccult; 4 points=gross bleeding[6]. Mice were sacrificed on day 9, and the weight of the spleen and the length of the colon were measured.

Histological Analysis: For HE stanning, the colon sections embedded in Paraffin were stained with hematoxylin and eosin, then analyzed by microscopy. For PAS and ROS staining, both were performed using PAS staining kit and ROS staining kit, following the manufacturer's protocol.

For immunohistochemistry and immunofluorescence assays, paraffin sections of colon sections were dried, deparaffinized, subjected to antigen retrieval and goat serum blockade. The primary antibodies, which included F4/80, CD86, and anti-MPO, were then incubated overnight at 4 °C. The next day, the specimens were rinsed with PBS three times and then incubated with corresponding fluorescent secondary antibody, followed by counterstained with 4′,6-diamidino-2-phenylindole (DAPI). Primary antibodies were shown in Table S1. Immunofluorescence images were captured using a fluorescence microscope.

Flow cytometry and cell sorting. The colons were isolated, cut into small pieces with micro-scissors, and mixed with collagenase II (1.5mg/mL, Worthington biochemical corporation), DNase I (0.5mg/mL, Worthington biochemical corporation) and elastase (0.25mg/mL, Worthington biochemical corporation) and enzymatically digested for 30min at 37°C. After digestion, the liquid was filtered by 40-μm cell strainers and centrifuged (300g for 5 min) in PBS. Single cell suspensions were incubated with live/dead-BV510 (1:1000 dilution, BD Biosciences, 564406) at room temperature for 10 min. Then cells were incubated with the following antibodies at 4 °C for 30 min: CD45-APC-750 (103154, BioLegend), CD11b-PreCP-Cy5.5 (101228, BioLegend), F4/80-PE (111703, BioLegend), and CD206-DAPI (141717, BioLegend). Flow cytometric analysis was performed on FACSAria™ III flow cytometer (BD Biosciences) and cells were sorted using a moflo Astrios EQ (Beckman Instruments, Inc). Data were analyzed with FlowJo 10 software (Tree Star).

Quantitative PCR Assay: Total RNA from colonic tissues was isolated with Trizol reagent (Solarbio, Beijing) and then transcribed into cDNA using cDNA Reverse Transcription kit (TransGen Biotech, Beijing). Relative expression of CCL2, CCL3, CCL5, CXCL8, CXCL10, CXCL12, Occludin, Claudin-7, ZO-1 and Cdh-1 were calculated using the comparative threshold cycle method (2−ΔΔCt

method) and all the values was normalized to the housekeeping β-actin gene. The primer sequences were listed in table S2.

Western Blotting: RAW264.7 cells and colon tissues samples were homogenized in lysis buffer with a complete protease inhibitor cocktail (Roche) and BCA assay was used to measure the concentration of protein. Proteins were subjected to sodium dodecyl sulfate polyacrylamide gel electrophoresis (SDS-PAGE), and then transferred to polyvinylidene fluoride (PVDF) membranes (Millipore), followed by incubation with primary antibodies against Raptor, HIF-1α, HK-2, PFKM, PKM2, LDHA, TNF-α, IL-1β, INF–γ, CD98, Arg-1, IL-10, Ym1 and GAPDH, and then incubation with secondary HRP-coupled antibodies. Quantitative analysis was performed with NIH Image J software.

Colon tissue ATP assay: The ATP content in the colon was detected using the Enhanced ATP Assay Kit (Beyotime Biotechnology, S0027) in accordance with the manufacturer protocol. Tissues were lysed in the ATP lysis buffer, and the protein in each sample was homogenized with a lysis buffer after protein quantification. The ATP contents were determined by measuring chemiluminescence with a luminometer plate reader (Promega Biotech Co. Ltd., Beijing, China).

Magnetic Resonance Imaging (MRI) of Colitis: 7-Tesla dedicated animal scanner (BioSpec 70/20 USR, Bruker, Ettlingen, Germany) utilizing a one-channel circular polarized volume coil (Bruker BioSpec MRI GmbH) was employed to observe the severity of colitis. Mice were anesthetized by isoflurane inhalation and their respiration was monitored and kept constant between 30-50 breaths/min during the entire examination using 2.0-2.5% isoflurane delivered by mixed oxygen and air (20%: 80%). Prone position was selected in the scanner, and the rectal temperature was monitored and maintained at 37 ± 0.5°C, and respiratory signals were also recorded (SA Instruments Inc., Stony Brook, NY).

The imaging protocol consisted of a T1-weighted spin echo and a T2-weighted turbo spin echo sequence. T1-weighted images were generated using a Fast Low Angle Shot (FLASH) sequence with parameters as follows: TE = 2.9 milliseconds, TR = 400 milliseconds, Flip Angle = 50°, FOV = 30 × 25 mm^2, slice interval = 1 mm, slices = 30, matrix = 192 × 160, average = 3. T2-weighted images were generated using a Tubro Rapid Acquisition with Relaxation Enhancement (RARE) sequence with parameters as follows: TE = 25 milliseconds, TR = 3000 milliseconds, FOV = 30 × 25 mm^2， slice interval = 1 mm, slices = 30, matrix = 256 × 214, average = 3. In order to avoid

respiratory artifacts, all sequences were triggered by respiratory gating. The MRI data analysis was accomplished by post processing software of the Biospin system (Paravision 6.0.1, Bruker, Ettlingen, Germany).

Biocompatibility Evaluation: Normal male C57BL/6 mice were divided into control group and Pd@M group, with three mice in each group and intravenously injected with saline and Pd@M (10 mg/kg), respectively. The mice were sacrificed at different times of administration (1, 7 and 30 days) to collect the heart, liver, spleen, lung, and kidney for HE staining. Meanwhile, the blood of the mice was collected for blood routine examination and biochemical analysis.

Supplementary figures: Figure S1- S18；Table S1-S2.

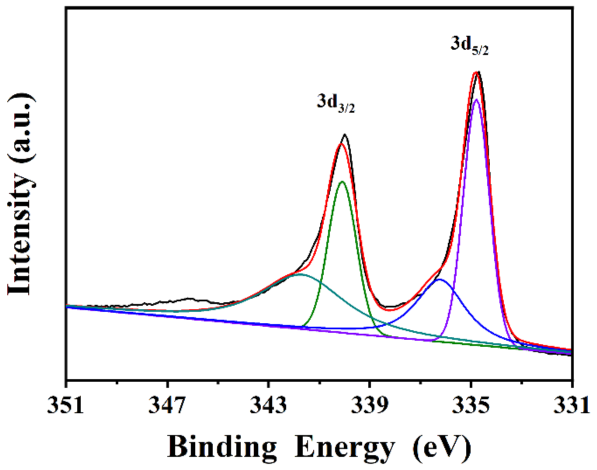

Figure S1. XPS spectrum of Pd.

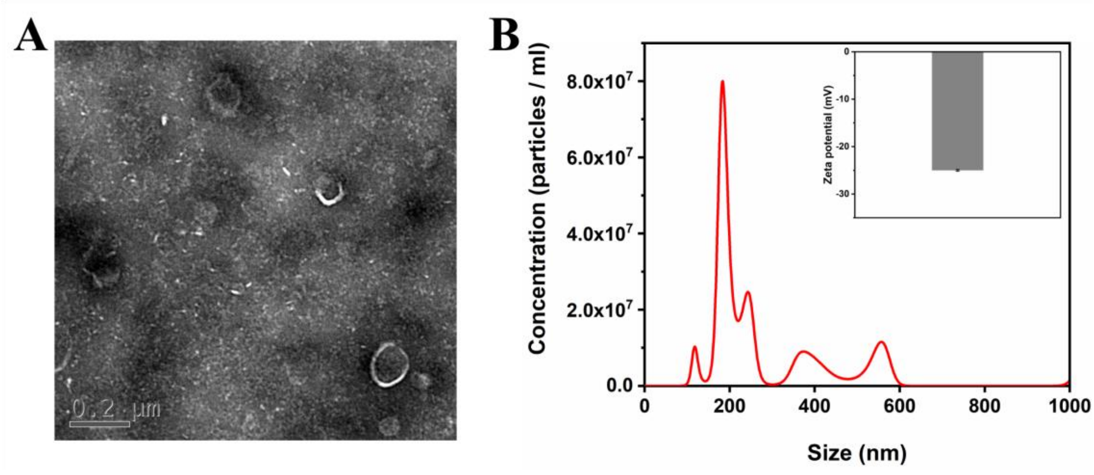

Figure S2. A) Typical TEM image of the obtained MEVs. B) The particle size and zeta potential of MEVs.

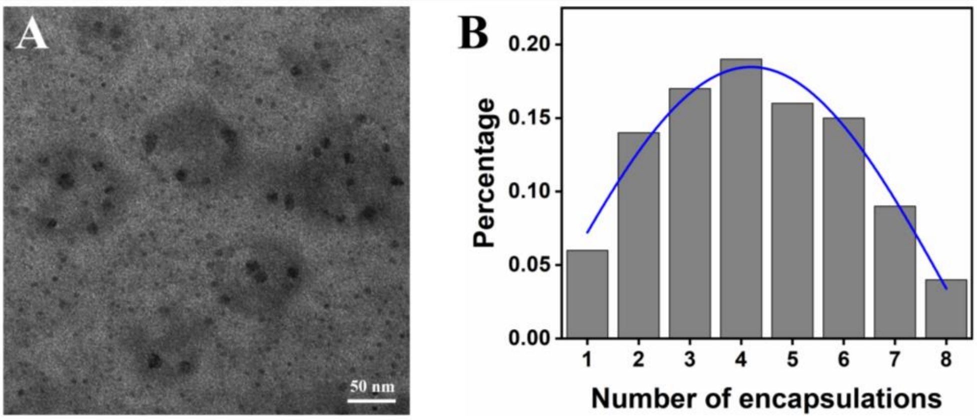

Figure S3. A) Typical TEM image of Pd@M after negative staining with *tungsten phosphate*. B) Percentage of number of Pd nanocubes encapsulated in a single MEV.

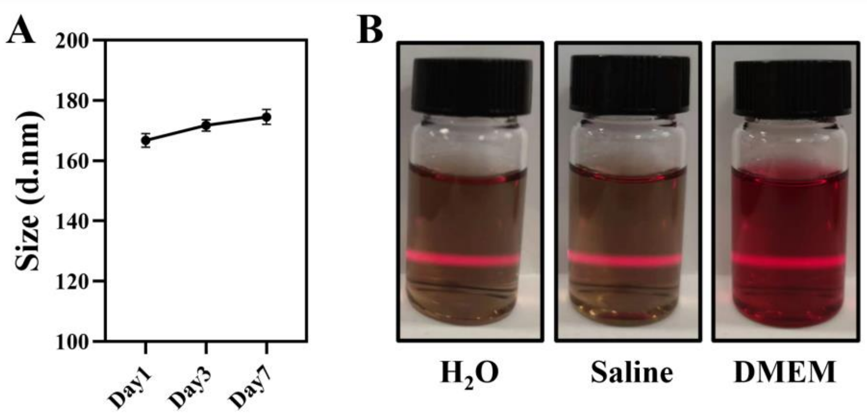

Figure S4. A) The mean particle sizes of Pd@M after 1, 3 and 7 days of storage (n = 3). B) Tyndall effect of Pd@M nanocubes in different solutions.

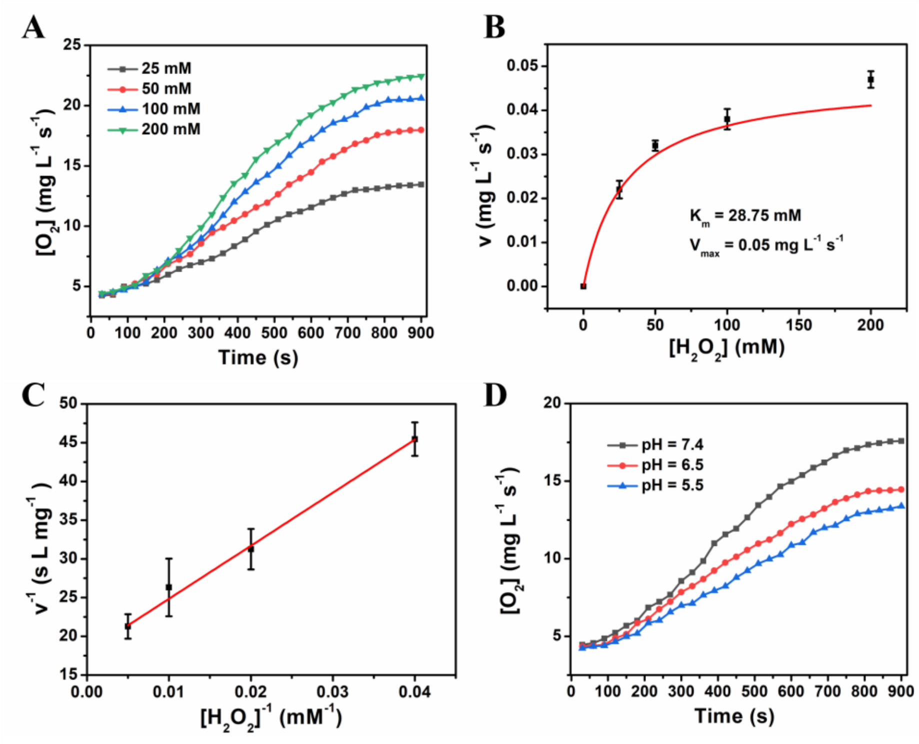

Figure S5. A) The O_2 generation changes of Pd@M reaction with different concentrations of H_2O_2 (25, 50, 100, 200 mM) at different durations. B) Michaelis-Menten kinetic analysis of catalase-like activities of Pd@M. Steady-state kinetic curves of Pd@M against H_2O_2 substrate and and (C) the corresponding double-reciprocal plots (n = 3). Data were expressed by mean ± SD. D) Changes of O_2 production from the reaction of Pd@M with H_2O_2 at different pH.

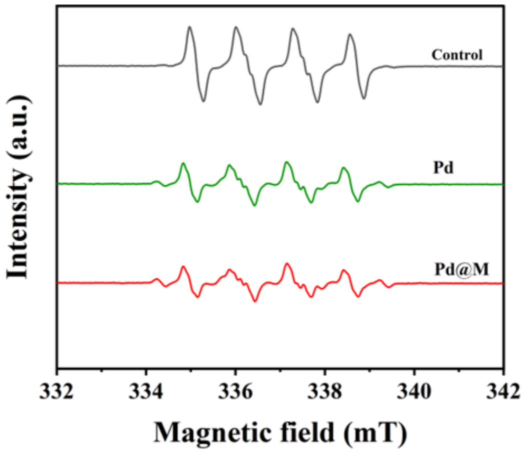

Figure S6. ESR curve of scavenging superoxide anion (•O2 ⁻) of Pd and Pd@M with DMPO as the spin trap.

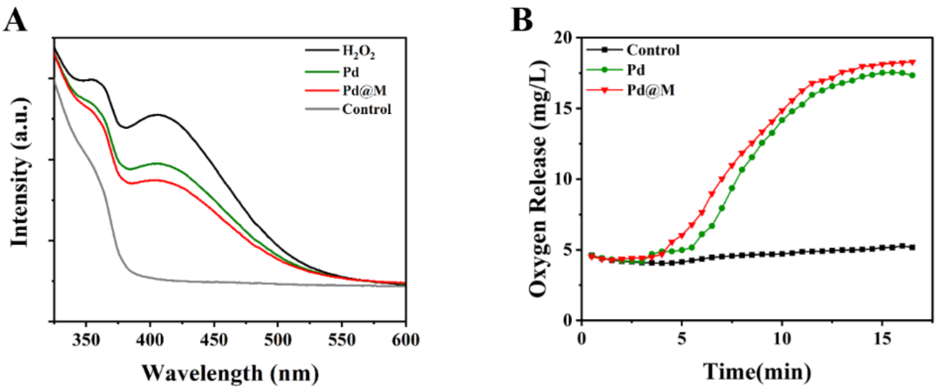

Figure S7. A) Evaluation of the CAT-like activities of Pd and Pd@M by UV-Vis utilizing a CAT kit. B) Oxygen concentration changes of Pd and Pd@M at different time points.

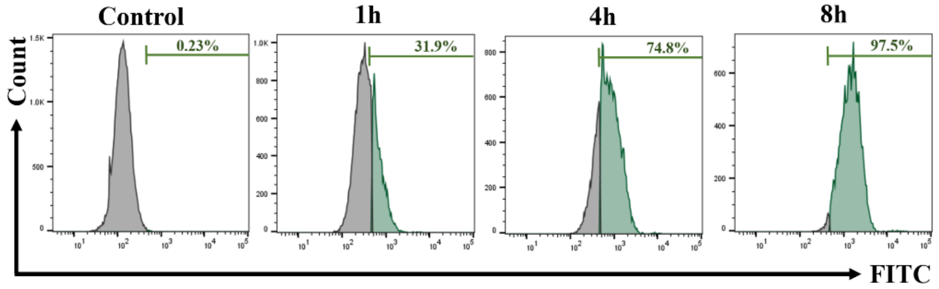

Figure S8. Flow cytometry analysis of RAW264.7 cells co-incubation with FITC-labeled Pd@M for 0, 1, 4 and 8 hours.

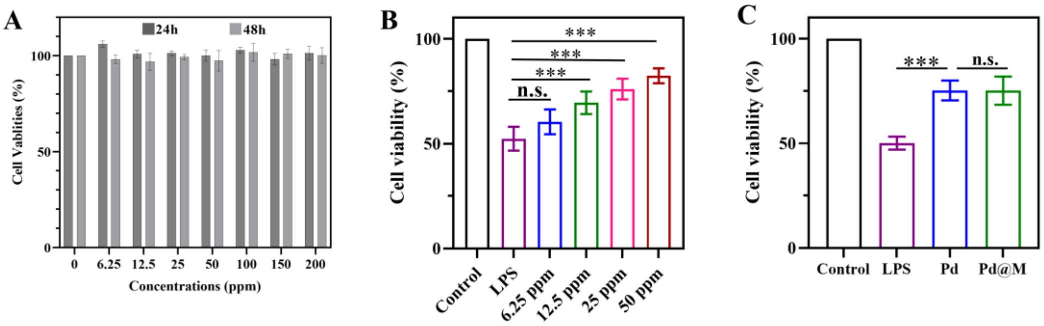

Figure S9. A) Cytotoxicity of Pd@M (μg/mL) on RAW264.7 cells. B) Relative cell viabilities of RAW264.7 cells after diverse treatments (n = 3). C) Cell viabilities of Pd and Pd@M treatments on LPS-stimulated RAW264.7 cells (n = 3). data are presented as mean ± SD. Statistically significant differences between groups were identified by one-way ANOVA with Tukey's post-hoc test. ***$p < 0.001$, n.s. implies no significance.

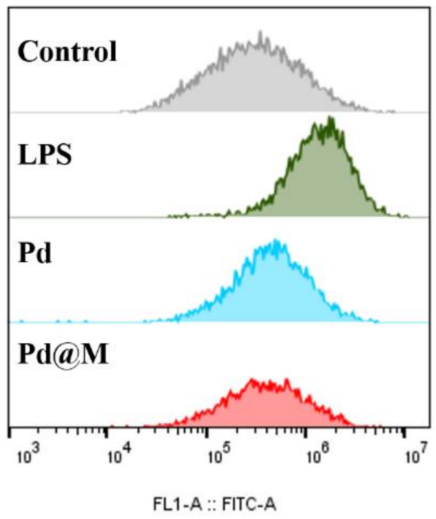

Figure S10. Detection of Pd and Pd@M reducing the ROS levels in LPS-stimulated RAW264.7 cells by flow cytometry *in vitro*.

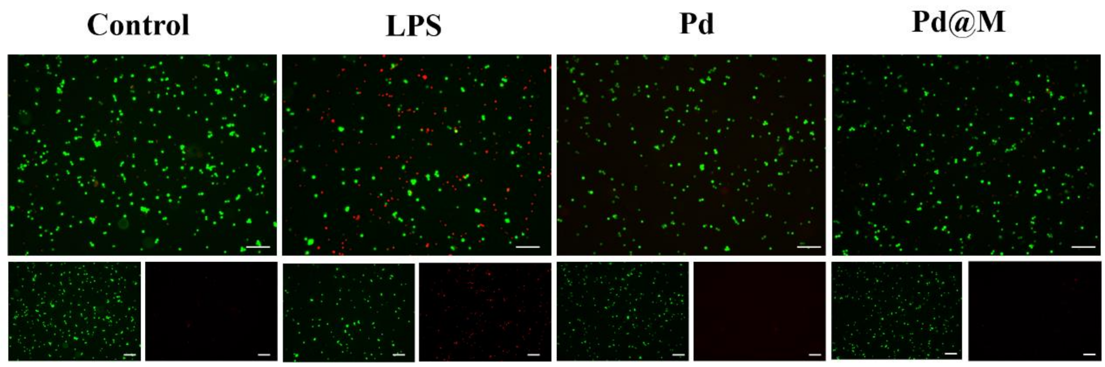

Figure S11. Detection of Pd and Pd@M effects on the ratio of live to dead cells in LPS-stimulated RAW264.7 cells by confocal microscopes *in vitro*. Bar = 200 μm.

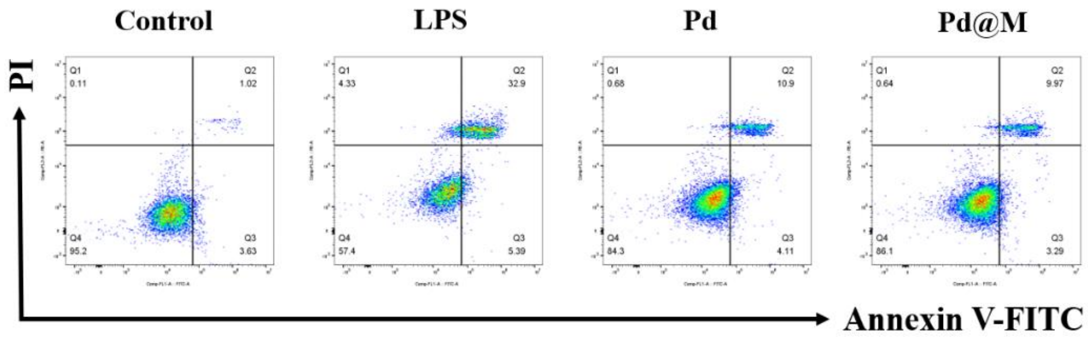

Figure S12. Detection of Pd and Pd@M reducing the apoptosis levels in LPS-stimulated RAW264.7 cells by flow cytometry *in vitro*.

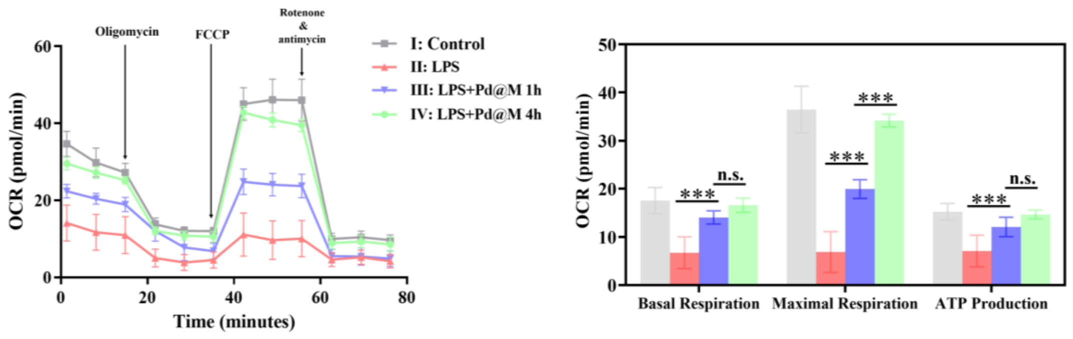

Figure S13. Oxygen consumption rate (OCR) plot from Seahorse Mito Stress assay performed on Raw264.7 cells with varied treatments. Bar chart showing the quantification of basal respiration, maximal respiration, and ATP production across the four groups (n = 5). For all panels, data are presented as mean ± SD. Statistically significant differences between groups were identified by one-way ANOVA with Tukey's post-hoc test. ***$p < 0.001$, n.s. implies no significant difference.

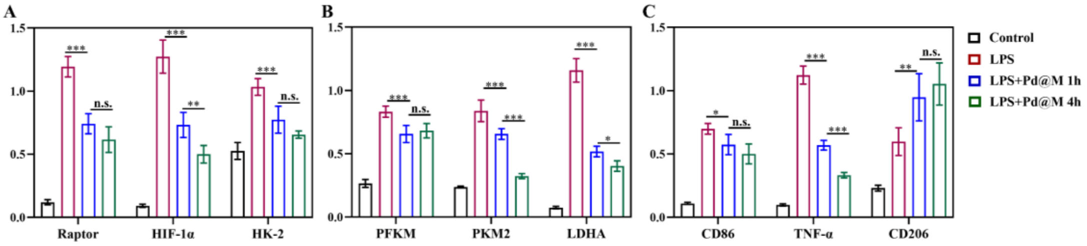

Figure S14. The semiquantitative data showing the protein expression of Raptor, HIF-1α, HK-2, PFKM, PKM2, LDHA, CD86, TNF-α and CD206 in LPS-stimulated RAW264.7 cells. (n = 5). Data are expressed as mean ± SD. Statistically significant differences between groups were identified by one-way ANOVA with Tukey's post-hoc test. *$p < 0.05$; **$p < 0.01$; ***$p < 0.001$, n.s. means no significant difference.

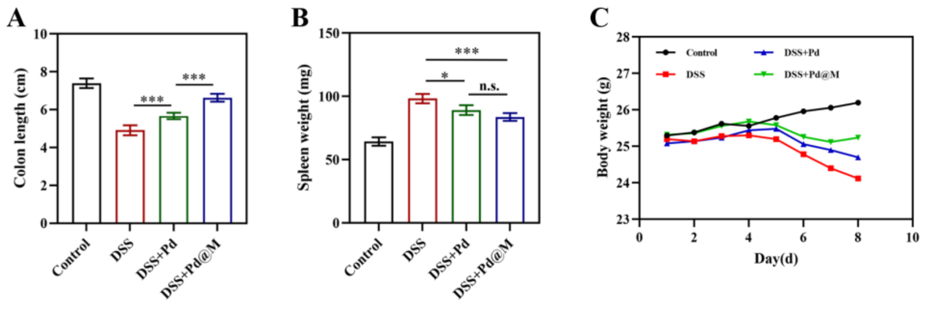

Figure S15. A-B) Statistical chart of the colon length and spleen weight of four groups on day 9 (n

= 5). C)The body weight changes of four groups during DSS-induced colitis and treatment periods (n = 5). Data are expressed as mean ± SD. Statistically significant differences between groups were identified by one-way ANOVA with Tukey's post-hoc test. *$p < 0.05$, ***$p < 0.001$, n.s. means not significant.

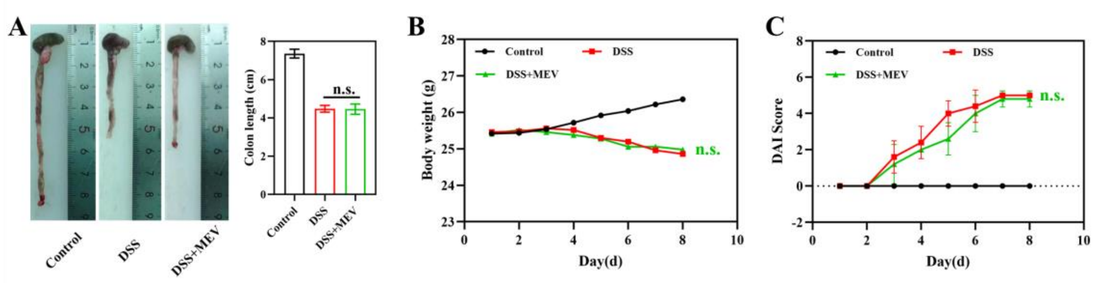

Figure S16. A) Typical macroscopic appearance of colons and statistical chart of the colon length (n = 5). B) The body weight changes of the control, DSS and MEVs treated groups (n = 5). C) The DAI score of mice of each group before day 9 (n = 5). Data are expressed as mean ± SD. Statistically significant differences between groups were identified by one-way ANOVA with Tukey's post-hoc test. n.s. means not significant.

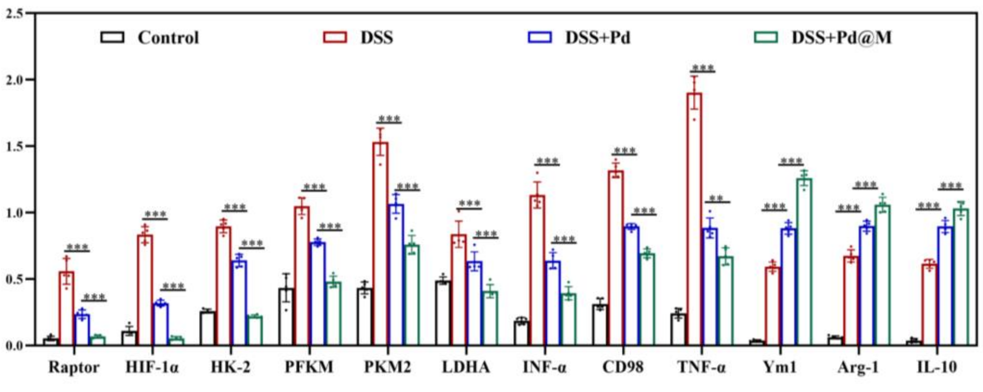

Figure S17. The semiquantitative data showing the protein expression of Raptor, HIF-1α, HK-2, PFKM, PKM2, LDHA, INF-γ, CD98, TNF-α, Ym1, Arg-1, and IL-10 in colon tissues from four groups on day 9 (n = 5). Data are expressed as mean ± SD. p-values are calculated using one-way ANOVA with Tukey's post-hoc test. **$p < 0.01$, ***$p < 0.001$.

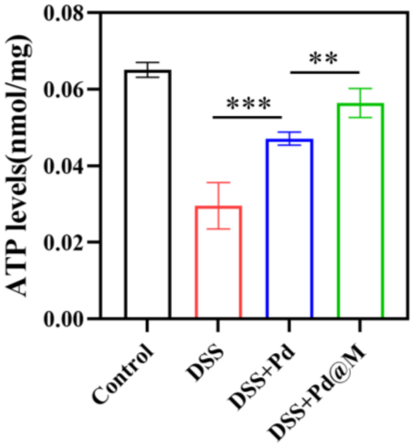

Figure S18. The ATP levels in colon tissues from four groups on day 9 (n = 6). Data are expressed as mean ± SD. *p*-values are calculated using one-way ANOVA with Tukey's post-hoc test. $**p < 0.01$, $***p < 0.001$.

Table S1.

List of the antibodies and kits used in animal experiments

Antibody specificity	Company	Cat. No.	Application	Dilution
**Mouse antibodies for WB**				
Raptor	ABclonal	A8992	WB	1:1000
HIF-1α	ABclonal	A11945	WB	1:1000
HK-2	ABclonal	A20829	WB	1:1000
PFKM	ABclonal	A3671	WB	1:1000
PKM2	Bioss	bs-0102M	WB	1:1000
LDHA	Bioss	bs-34202R	WB	1:1000
GAPDH	HuaBio	ET1601-4	WB	1:5000
CD86	ABclonal	A19026	WB	1:1000
TNF-α	ABclonal	A22227	WB	1:1000
CD206	ABclonal	A11192	WB	1:1000
CD98	ABclonal	A5702	WB	1:1000
INF-γ	ABclonal	A21081	WB	1:1000
Ym1	Abcam	Ab192029	WB	1:20000
Arg-1	ABclonal	A1847	WB	1:1000
IL-10	ABclonal	A12255	WB	1:1000
β-actin	ABclonal	AC026	WB	1:50000
CD9	ABclonal	A19027	WB	1:2000
CD63	CST	52090S	WB	1:2000
CD44	Servicebio	GB112054	WB	1:2000
Mac-1	CST	17800S	WB	1:2000
P-selectin	Proteintech	60322-1-Ig	WB	1:2000
ICAM-1	CST	67836S	WB	1:2000
**Secondary antibodies for WB**				
HRP-Goat anti Rabbit	Jackson	111-035-003	WB	1:5000
HRP-Goat anti mouse	Proteintech	SA00001-1	WB	1:5000
**Mouse antibodies for IF**				
F4/80	CTS	70076	IF	1:200
CD86	Abclonal	A2353	IF	1:100
DAPI	Invitrogen	P36931	IF	/
MPO	Abcam	Ab208670	IHC	1:1000
**Secondary antibodies for IF**				
HRP-Goat anti Rabbit	Abcam	Ab205718	IF	1:2000
HRP-Goat anti Rabbit	Abcam	Ab205718	IHC	1:2000

Other kits				
ROS	Beyotime	S0033	/	
PAS	Abcam	ab245876	/	
ATP	Beyotime	S0027		

Table S2.

引物名称	序列（5' to 3'）
Mouse GAPDH F	GCAGTGGCAAAGTGGAGATT
Mouse GAPDH R	TCTCCATGGTGGTGAAGACA
Mouse Cdh 1 F	GGTCATCAGTGTGCTCACCTCT
Mouse Cdh 1R	GCTGTTGTGCTCAAGCCTTCAC
Mouse Claudin 7 F	CCCCTCCACTTCTTTGGGTAG
Mouse Claudin 7 R	GGGCGACCCTACAGTAAAGC
Mouse occludin F	TGGCAAGCGATCATACCCAGAG
Mouse occludin R	CTGCCTGAAGTCATCCACACTC
Mouse ZO-1 F	GTTGGTACGGTGCCCTGAAAGA
Mouse ZO-1 R	GCTGACAGGTAGGACAGACGAT
Mouse CCL2 F	GCTACAAGAGGATCACCAGCAG
Mouse CCL2 R	GTCTGGACCCATTCCTTCTTGG
Mouse CCL3 F	ACTGCCTGCTGCTTCTCCTACA
Mouse CCL3 R	ATGACACCTGGCTGGGAGCAAA
Mouse CCL5 F	CCTGCTGCTTTGCCTACCTCTC
Mouse CCL5 R	ACACACTTGGCGGTTCCTTCGA
Mouse Cxcl10 F	ATCATCCCTGCGAGCCTATCCT
Mouse Cxcl10 R	GACCTTTTTTGGCTAAACGCTTTC
Mouse Cxcl2 F	CATCCAGAGCTTGAGTGTGACG
Mouse Cxcl2 R	GGCTTCAGGGTCAAGGCAAACT
Mouse Cxcl8 F	ATATTGGCAACGACCCCCAG
Mouse Cxcl8 R	GATGAAGGCCTGGAACGTCA

[1] M. Jin, H. Liu, H. Zhang, Z. Xie, J. Liu, Y. Xia, *Nano Research* **2011**, *4*, 83.
[2] R. Li, Y. He, Y. Zhu, L. Jiang, S. Zhang, J. Qin, Q. Wu, W. Dai, S. Shen, Z. Pang, J. Wang, *Nano Lett* **2019**, *19*, 124.
[3] X. Ye, L. Xia, H. Yang, J. Xu, T. Liu, L. Wang, S. Zhang, Y. Chen, D. Du, W. Feng, *Mater Today* **2023**.
[4] B. Plitzko, S. Loesgen, *Bio Protoc* **2018**, *8*, e2850.
[5] S. Wirtz, V. Popp, M. Kindermann, K. Gerlach, B. Weigmann, S. Fichtner-Feigl, M. F. Neurath, *Nat Protoc* **2017**, *12*, 1295.
[6] F. da Costa Gonçalves, N. Schneider Nunes, H. Mello, E. Pandolfi Passos, L. Meurer, E. Cirne-Lima, A. Paz, *Acta Scientiae Veterinari* **2013**, *41*.
